# Genomic characterization of peste des petits ruminants vaccine seed “45G37/35-k”, Russia

**DOI:** 10.1186/s13567-022-01099-w

**Published:** 2022-10-08

**Authors:** Olivier Kwiatek, Geneviève Libeau, Samia Guendouz, Chloé Corbanini, Andrey Gogin, Andrey Lunitsin, Irina Sindryakova, Denis Koblasov, Arnaud Bataille

**Affiliations:** 1 CIRAD, UMR ASTRE, 34398 Montpellier, France; 2 ASTRE, University of Montpellier, CIRAD, INRAE, Montpellier, France; 3 Federal Research Center for Virology and Microbiology, 601125 Volginsky, Russia

## Abstract

Production of peste des petits ruminants (PPR) vaccines in Russia is based on two attenuated virus strains (“45G37/35-k” and “ARRIAH”) of common origin. Here, the identity of the strain PPRV/45G37/35-k was investigated using a full genome, Illumina deep sequencing approach. Phylogenomic analysis showed that PPRV/45G37/35-k belongs to the same lineage as the widely used PPRV vaccine strain Nigeria/75/1 (lineage II). However, 248 nucleotide differences separate the genomes of these vaccine strains, indicating that the PPRV vaccine strains produced in Russia are new strains not yet recognised by the World Organization for Animal Health (WOAH). Detailed information on the safety and efficacy of these vaccines should be provided to the WOAH before further national and international distribution.

**Supplementary Information:**

The online version contains supplementary material available at 10.1186/s13567-022-01099-w.

## Introduction, methods and results

Peste des petits ruminants (PPR) is a highly contagious viral disease of domestic and wild small ruminants with a widespread distribution spanning Africa, apart from the southern countries, the Arabian Peninsula, the Middle East and Central and Southern Asia [1–3]. Europe and Russia are free from the disease, but outbreaks of PPR in Georgia in 2016 [4], in Mongolia in 2016–2017 and in 2021–2022 [5, 6], and an incursion in Bulgaria in 2018 [7] highlight the threat of emergence in this part of the globe, and notably in Russia where susceptible animals traditionally share high mountain pastures with animals from neighbouring countries.

As a viral disease, no specific treatment exists for PPR, and the vaccination of small ruminants is the only effective way of controlling this disease. PPR is now targeted for global eradication through vaccination, in line with the PPR Global Control and Eradication Strategy (GCES) devised by World Organization for Animal Health (WOAH) and the Food and Agriculture Organization of the United Nations (FAO) [8]. The WOAH provides science-based standards, guidelines and recommendations to control the disease in animals and to prevent the spread of the disease through trade; the WOAH also provides intergovernmental standards for the diagnosis of the disease and the quality of vaccines for use in animals. Thus extensive quality control of the PPR vaccines available, according to requirements described in the WOAH Terrestrial Manual, is a prerequisite to ensure the safety and efficacy of the vaccination campaigns planned in the eradication program [1, 9]. The most widely used, WOAH-accepted PPR vaccine is the attenuated PPRV vaccine strain Nigeria/75/1, of which the Master Seed is made available to vaccine producers by CIRAD, one of its developers [10]. Other PPRV attenuated vaccine strains accepted by the WOAH are used in some regions [1, 11].

Two institutions in Russia are authorized to produce PPR vaccine: the Federal Research Center for Virology and Microbiology (FRCVM) and the Federal Governmental Budgetary Institution “Federal Centre for Animal Health” (ARRIAH). Each institution holds different attenuated virus strains called “45G37/35-k” (for FRCVM), and “ARRIAH” (for ARRIAH), both originating from the same “45G35” strain attenuated in the 1980s in the USSR after 35 passages in lamb kidney cells. The strain “45G37/35-k” was obtained at FRCVM after passages in lamb kidney, lamb testes, and Saiga kidney primary cells. Detailed information on the characteristics of this vaccine, its production and quality control are described in two patents RU 2 325 185 C1 [12] and RU 2 284 193 C1 [13]. However, its genetic identity has never been fully explored and compared with other vaccines strains used in the vaccination campaigns. The additional passage history of the “ARRIAH” strain is not known.

Recent advances in genotypic characterization methods have allowed the use of increasingly refined techniques to explore PPR virus (PPRV) genetic diversity, and therefore to monitor the emergence and fixation of new genetic variants related to cell passages [14]. Such methodology has been used previously to follow the process of attenuation of the PPRV vaccine strain Nigeria/75/1, allowing the identification of only 18 mutations distributed across the PPRV genome and differentiating the vaccine strain from the wild type strain originally isolated and attenuated through cellular passages [14].

The library preparation and high throughput Illumina sequencing methods described in [14] were used on the PPRV/45G37/35-k strain. Briefly, cDNA synthesised from the vaccine RNA was amplified by PCR using five overlapping fragments of 3-4 kb. Purified fragments were pooled in equal concentration to prepare an Illumina sequencing library using the Nextera kit. Sequencing was performed on an Illumina MiSeq machine at the AGAP sequencing platform (CIRAD, France).

**Figure 1:**
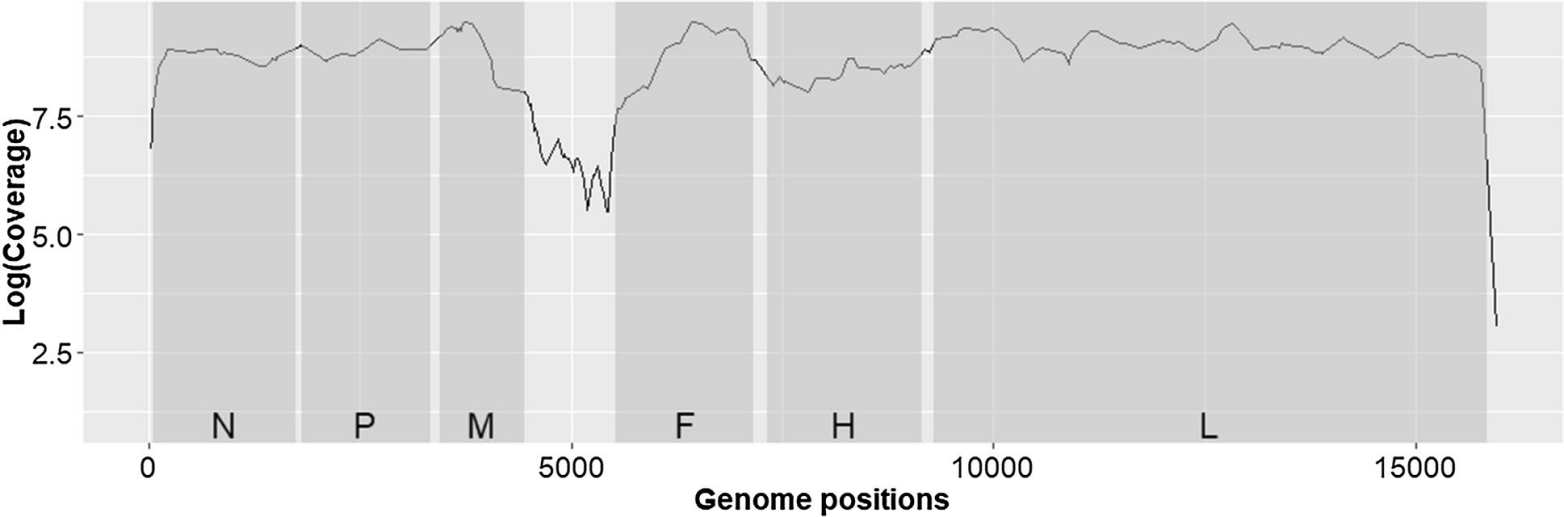
**Depth (number of reads in log scale) obtained by Illumina sequencing on each gene region of the PPRV PPRV/45 G37/35-k vaccine strain genome**. Grey areas represent the coding regions (CDS) of the following viral genes: N (nucleocapsid), P (phosphoprotein), M (matrix), F (fusion), H (haemagglutinin) and L (polymerase).

A total of 3 865 602 million reads were obtained (NCBI Sequence Read Archive accession number: SAMN27763131) and analysed using a homemade bioinformatics pipeline [15], with the genome of the Nigeria/75/1 vaccine seed (GenBank n KY628761) as reference for the alignment of reads, to determine the consensus sequence within the raw data of the sample, as well as to detect single nucleotide variations (SNVs) within the population. Calling of variants was limited to positions covered with a minimum of 100 reads and for variants present with > 1% frequency. A total of 99.6% of the genome was covered with an average depth of 7050 reads/position (Figure 1). Only the bases overlapping with primers situated at the extremities of the genome could not be sequenced.

The genome sequence of the PPRV/45G37/35-k strain (Genbank accession number: ON207131) showed 248 nucleotide differences from the PPRV Nigeria/75/1 vaccine master seed (Additional file 1, Figure 2). Dissimilarities were found throughout the genome, with a majority of them present in over 99% in the reads covering the position, suggesting that they are fixed in the population. Among 248 mutations, 40 were non-synonymous (Additional file 2). Out of the 18 nucleotide positions differentiating the attenuated PPRV Nigeria/75/1 vaccine strain from its wild original strain, 17 observed in the PPRV/45G37/35-k genome corresponded to the variant identified in wild Nigeria/75/1 strain (Additional file 1). The last one (position 5) could not be sequenced.

**Figure 2:**
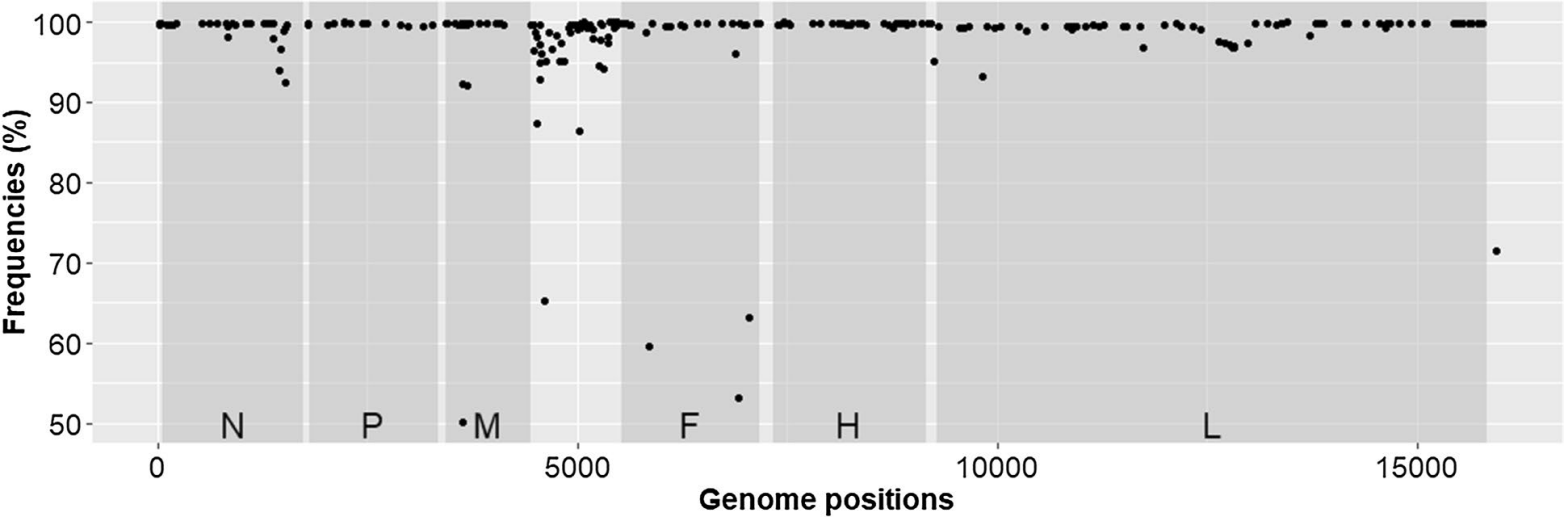
**Distribution of single nucleotide variant (SNV) frequencies according to the PPRV/45 G37/35-k vaccine strain genome positions in comparison to the genome of the PPRV Nigeria/75/1 vaccine strain**. Grey areas represent the coding regions (CDS) of the following viral genes: N (nucleocapsid), P (phosphoprotein), M (matrix), F (fusion), H (haemagglutinin) and L (polymerase).

The PPRV/45G37/35-k genome was aligned with published PPRV genomes using clustalW as implemented in Geneious v10.2.6. From this dataset of aligned sequences, a phylogenomic tree was built using the Maximum Likelihood method with the MEGA software following the parameters of the best substitution models obtained, chosen based on the lowest Bayesian Information Criterion (BIC) score. The model used is the GTR (General Time Reversible) with discrete Gamma distribution (+ G) and sites that are evolutionarily invariable (+ I). A bootstrap analysis of 1000 replicates was implemented to evaluate the robustness of the phylogenomic relationships.

**Figure 3:**
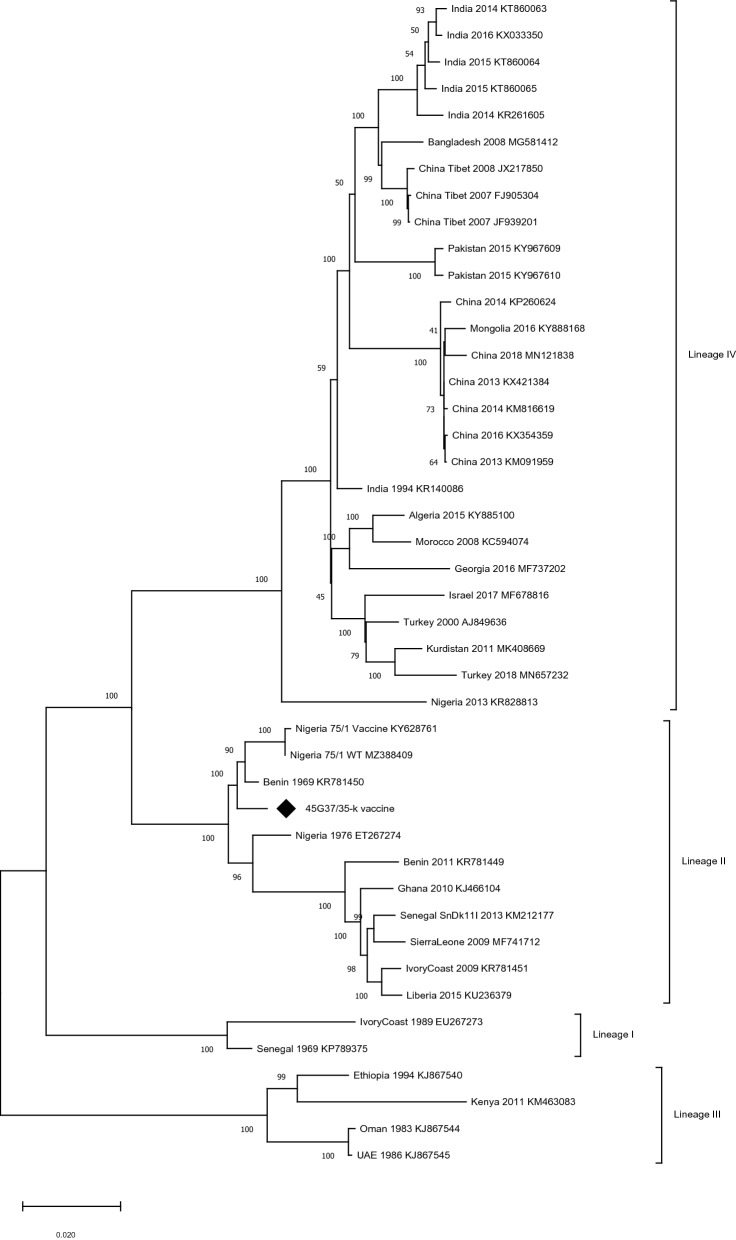
**Peste des petits ruminants virus full genome phylogenetic analysis**. The phylogenomic tree is constructed using a maximum likelihood method and aligned PPRV genomes. Numbers at the nodes of the tree indicates bootstrap support values obtained from 1000 replicates. Accession number of publically available genomes are indicated in the tree. The genome obtained during this study is indicated with an arrow.

The phylogenomic tree obtained showed that the position of the PPRV/45G37/35-k strain falls within a clade that includes the old strains of the PPRV lineage II, but was phylogenetically distinct from both the Nigeria/75/1 vaccine and wild type strains (Figure 3).

## Discussion

The characterization of the full genome of the PPRV vaccine strain PPRV/45G37/35-k held by the Federal Research Center for Virology and Microbiology (FRCVM) showed that this vaccine is significantly different from the Nigeria/75/1 vaccine strain. Its position within the old strains of PPRV lineage II in the phylogenomic tree confirms that it was produced decades ago, possibly at the same time as Nigeria/75/1, but that it derived from a different field isolate.

Previous genomic comparison of the Nigeria/75/1 vaccine and wild type strains showed that 18 mutations or less were important for the attenuation of this strain [14]. None of these mutations are present in the genome of PPRV/45G37/35-k. In addition, a high number (40) of non-synonymous mutations differentiated the two strains across all viral proteins. It is hard to assess the importance of each mutation on viral protein functions without the genome of the wild type strain at the origin of PPRV/45G37/35-k. Still, these results clearly suggest that the process of attenuation was different for this vaccine compared to Nigeria/75/1. The Nigeria/75/1 [10] and the Sungri/96 [11] PPRV vaccine strains were attenuated by serial passage (*n* = 75 and 59, respectively) in Vero cells. Different cell types were used for the attenuation of PPRV/45 G37/35-k, and it is probable that it generated different selection pressure on the virus. However, it is also possible that random accumulation of mutations during cell passages led to a different pathway towards the loss of virulence [16].

The use of recognized, high-quality and well-characterized vaccines is critical for the global PPR eradication effort. The Nigeria/75/1 [10] and the Sungri/96 [11] PPRV vaccine strains, widely used throughout the world in the PPR eradication program, confer long-lasting robust immunity in sheep and goats (at least up to 3 years), and have shown until now to be safe in a large variety of small ruminant breeds of Europe, Africa and Asia. The Nigeria/75/1 vaccine has been shown to resist to virulence reversion by 3 back passages in sheep [10]. The safety and efficacy of both vaccines strains have been further documented recently in studies carried out to compare the immunogenicity and protective efficacy of PPR vaccines following nasal or subcutaneous vaccine delivery in target species [17–19]. However, such safety and efficacy information is not published for the PPRV/45G37/35-k strain, except for what is included in the Russian patent.

The PPRV/ARRIAH strain is likely closely related to PPRV/45G37/35-k, but genomic characterization would be necessary to confirm this hypothesis. In all cases, no information regarding the validation of these PPR vaccine strains has been published and peer-reviewed by the international community. The fact that they are different from the widely used Nigeria/75/1 vaccine does not prejudge the efficacy of the PPRV/45G37/35-k and ARRIAH vaccines themselves. In case the FRCVM or ARRIAH intend to include this strain in the list of PPR vaccines recommended for the eradication program, WOAH would need to receive all information ensuring its safety and efficacy before recognizing it for use in vaccination campaigns planned in the eradication program.

## Supplementary Information

**Additional file 1.** **Position and frequency of the 248 nucleotide differences separating the PPR vaccine strains Nigeria/75/1 (Nig75/1) and 45G37/35-k (FRCVM).** Nucleotide positions corresponding to mutations associated to the attenuation of Nigeria/75/1 are in bold [14].

**Additional file 2.** **Distribution of nucleotide and amino acid differences separating the PPR vaccine strains Nigeria/75/1 (Nig75/1) and 45G37/35-k (FRCVM).**

## Data Availability

The genetic data generated and analysed during the current study are available in the NCBI GenBank (Accession Number: ON207131) and Sequence Read Archive repository (accession number: SAMN27763131).
