# Supplementary material for: Genomic characterization of peste des petits ruminants vaccine seed “45G37/35-k”, Russia
Source: Vet Res. 2022 Oct 8;53:79. doi: 10.1186/s13567-022-01099-w (PMC9548208; doi:10.1186/s13567-022-01099-w)
Supplement: Supplementary file 1 — Additional file 1. Position and frequency of the 248 nucleotide differences separating the PPR vaccine strains Nigeria/75/1 (Nig75/1) and 45G37/35-k (FRCVM). Nucleotide positions corresponding to mutations associated to the attenuation of Nigeria/75/1 are in bold [14]. [file 13567_2022_1099_MOESM1_ESM.docx]

**Additional file 1 Position and frequency of the 248 nucleotide differences separating the PPR vaccine strains Nigeria/75/1 (Nig75/1) and 45 G37/35-k (FRCVM).** Nucleotide positions corresponding to mutations associated to the attenuation of Nigeria/75/1 are in bold [12].

| Nucleotide differences between the two vaccines | | | | |
| --- | --- | --- | --- | --- |
| FRCVM | Nig75/1 | Genome Position | Coverage (nb of reads) | Variant Frequency |
| C | A | 24 | 908 | 99.8% |
| A | T | 26 | 1192 | 99.7% |
| **A** | **G** | **36** | **1918** | **99.9%** |
| G | T | 113 | 4947 | 99.7% |
| G | A | 158 | 5972 | 99.7% |
| C | T | 176 | 6133 | 99.7% |
| **T** | **C** | **221** | **7363** | **99.9%** |
| A | G | 520 | 7055 | 99.8% |
| G | A | 620 | 7277 | 99.9% |
| C | A | 707 | 7369 | 99.8% |
| T | C | 815 | 7425 | 99.9% |
| G | A | 830 | 7066 | 98.2% |
| T | C | 836 | 6814 | 99.4% |
| C | A | 893 | 6895 | 99.8% |
| G | A | 929 | 6853 | 99.7% |
| A | G | 1049 | 6648 | 99.9% |
| C | T | 1097 | 6359 | 99.8% |
| C | T | 1262 | 5435 | 99.8% |
| G | A | 1304 | 5168 | 99.9% |
| T | C | 1358 | 5316 | 99.8% |
| T | C | 1378 | 5322 | 97.9% |
| A | G | 1380 | 5308 | 99.9% |
| T | C | 1445 | 5868 | 94.0% |
| T | C | 1459 | 6137 | 96.6% |
| T | C | 1497 | 6037 | 98.8% |
| **T** | **C** | **1513** | **6351** | **92.5%** |
| C | A | 1525 | 6317 | 99.2% |
| G | C | 1536 | 6630 | 99.7% |
| T | C | 1786 | 7938 | 99.6% |
| T | C | 1791 | 8043 | 99.8% |
| **C** | **T** | **1795** | **8132** | **99.9%** |
| G | A | 2027 | 6366 | 99.7% |
| A | T | 2102 | 5827 | 99.8% |
| C | T | 2106 | 5930 | 99.9% |
| G | A | 2218 | 6475 | 100.0% |
| A | G | 2221 | 6458 | 99.9% |
| G | C | 2289 | 6819 | 99.8% |
| A | G | 2439 | 6512 | 99.8% |
| C | T | 2469 | 7028 | 99.9% |
| G | A | 2487 | 6970 | 99.8% |
| A | T | 2720 | 9398 | 99.9% |
| A | T | 2895 | 7905 | 99.7% |
| A | G | 2982 | 7378 | 99.5% |
| **G** | **A** | **3158** | **7390** | **99.5%** |
| T | C | 3279 | 7392 | 99.7% |
| G | A | 3433 | 9550 | 99.9% |
| C | T | 3458 | 9951 | 99.9% |
| G | T | 3539 | 11831 | 99.9% |
| G | A | 3545 | 11696 | 99.8% |
| A | G | 3572 | 11938 | 99.7% |
| C | T | 3620 | 11589 | 99.9% |
| T | C | 3630 | 11659 | 99.7% |
| T | C | 3639 | 11558 | 92.3% |
| T | A | 3641 | 11427 | 50.2% |
| G | T | 3647 | 11000 | 99.8% |
| G | A | 3650 | 11086 | 99.9% |
| C | T | 3677 | 11734 | 99.9% |
| TT | CC | 3691-3692 | 10793 -> 10892 | 92.1% -> 92.3% |
| **A** | **G** | **3694** | **10937** | **99.7%** |
| G | A | 3701 | 11822 | 99.9% |
| T | C | 3731 | 13235 | 99.8% |
| C | T | 3822 | 12765 | 99.9% |
| C | T | 3930 | 9785 | 99.8% |
| T | A | 4022 | 6613 | 99.9% |
| **A** | **C** | **4046** | **5879** | **99.9%** |
| A | G | 4079 | 3955 | 99.9% |
| G | A | 4125 | 3344 | 99.6% |
| A | G | 4127 | 3396 | 99.6% |
| T | C | 4451 | 3011 | 99.7% |
| T | C | 4472 | 2869 | 96.4% |
| A | G | 4478 | 2775 | 99.7% |
| C | T | 4486 | 2516 | 99.6% |
| T | C | 4492 | 2473 | 98.6% |
| T | C | 4508 | 2284 | 98.2% |
| A | C | 4512 | 2345 | 87.4% |
| T | C | 4548 | 1364 | 94.9% |
| TC | CT | 4550-4551 | 1335 -> 1337 | 92.9% -> 93.0% |
| C | T | 4553 | 1335 | 97.2% |
| **G** | **A** | **4559** | **1413** | **99.6%** |
| CT | AC | 4570-4571 | 1428 -> 1430 | 96.0% -> 96.1% |
| T | - | 4607 | 1127 | 65.3% |
| T | C | 4623 | 934 | 95.1% |
| G | A | 4653 | 771 | 98.6% |
| T | A | 4698 | 642 | 96.6% |
| C | A | 4759 | 779 | 98.3% |
| T | C | 4789 | 925 | 95.1% |
| T | C | 4804 | 966 | 97.4% |
| G | A | 4835 | 1108 | 95.1% |
| C | T | 4897 | 761 | 99.3% |
| T | C | 4911 | 809 | 98.6% |
| A | G | 4919 | 770 | 99.7% |
| A | G | 4971 | 743 | 99.7% |
| C | A | 5001 | 645 | 99.1% |
|  | C | 5014 | 546 | 86.4% |
| A | G | 5040 | 673 | 99.3% |
| A | G | 5047 | 710 | 99.9% |
| G | A | 5085 | 749 | 100.0% |
| A | G | 5095 | 697 | 99.9% |
| C | T | 5097 | 695 | 99.7% |
| GC | AT | 5115-5116 | 609 -> 612 | 99.3% |
| A | G | 5143 | 455 | 99.6% |
| C | T | 5176 | 276 | 98% |
| C | T | 5178 | 247 | 99% |
| TA | CC | 5258-5259 | 522 -> 548 | 94.6% -> 94.7% |
| C | T | 5271 | 515 | 97.7% |
| C | T | 5273 | 522 | 99.8% |
| C | G | 5292 | 561 | 99.6% |
| T | C | 5312 | 622 | 94.1% |
| G | A | 5371 | 376 | 97.3% |
| T | C | 5374 | 361 | 98.1% |
| C | T | 5379 | 340 | 100.0% |
| G | A | 5409 | 241 | 100.0% |
| G | A | 5429 | 239 | 100.0% |
| C | T | 5437 | 286 | 99.3% |
| G | A | 5445 | 383 | 100.0% |
| C | T | 5451 | 441 | 99.5% |
| C | A | 5466 | 700 | 99.9% |
| C | T | 5471 | 720 | 100.0% |
| C | T | 5506 | 1350 | 99.9% |
| G | A | 5546 | 2102 | 99.8% |
| G | A | 5577 | 2165 | 99.9% |
| **A** | **G** | **5627** | **2543** | **99.6%** |
| T | C | 5639 | 2709 | 99.7% |
| A | G | 5645 | 2711 | 99.7% |
| G | A | 5819 | 3272 | 98.7% |
| C | T | 5854 | 3453 | 59.5% |
| G | A | 5894 | 3277 | 99.9% |
| T | C | 5897 | 3251 | 99.8% |
| T | C | 6053 | 6025 | 99.4% |
| G | T | 6104 | 7375 | 99.5% |
| A | C | 6230 | 8389 | 99.6% |
| A | G | 6275 | 8489 | 99.4% |
| **A** | **C** | **6422** | **13107** | **99.9%** |
| C | T | 6428 | 13271 | 99.8% |
| T | C | 6545 | 12933 | 99.8% |
| T | G | 6713 | 10445 | 99.8% |
| **A** | **G** | **6846** | **11555** | **99.8%** |
| A | G | 6878 | 11336 | 96.1% |
| A | G | 6912 | 11441 | 53.1% |
| C | A | 6941 | 11314 | 99.8% |
| A | G | 6978 | 10670 | 99.6% |
| G | A | 7001 | 10014 | 99.7% |
| G | A | 7049 | 9080 | 63.2% |
| G | A | 7130 | 5940 | 99.9% |
| C | T | 7168 | 5929 | 99.9% |
| **A** | **C** | **7384** | **3456** | **99.7%** |
| A | G | 7405 | 3734 | 99.7% |
| G | A | 7451 | 3886 | 99.9% |
| C | T | 7463 | 4168 | 100.0% |
| G | A | 7511 | 3683 | 99.8% |
| A | G | 7523 | 3832 | 99.7% |
| G | A | 7802 | 3031 | 99.9% |
| G | A | 7899 | 4043 | 99.9% |
| A | C | 8041 | 4046 | 99.9% |
| G | A | 8108 | 3956 | 99.8% |
| G | A | 8159 | 3997 | 99.8% |
| G | A | 8195 | 4276 | 99.7% |
| A | T | 8258 | 5833 | 99.7% |
| C | T | 8264 | 5950 | 99.9% |
| T | G | 8272 | 6191 | 99.9% |
| C | A | 8339 | 6074 | 99.9% |
| T | A | 8349 | 5871 | 99.8% |
| A | G | 8390 | 5089 | 99.8% |
| T | G | 8432 | 5099 | 99.7% |
| **C** | **T** | **8645** | **4903** | **99.9%** |
| G | T | 8699 | 4444 | 99.7% |
| T | C | 8752 | 5029 | 99.3% |
| G | A | 8798 | 5274 | 99.9% |
| **C** | **A** | **8829** | **5136** | **99.8%** |
| A | G | 8885 | 5427 | 99.8% |
| T | C | 8921 | 5474 | 99.8% |
| AA | CG | 8927 | 5062 -> 5065 | 99.7% |
| A | G | 8930 | 5135 | 99.7% |
| T | C | 9002 | 5206 | 99.8% |
| T | C | 9110 | 6387 | 99.9% |
| A | G | 9177 | 7367 | 99.8% |
| T | C | 9198 | 7278 | 99.9% |
| G | C | 9216 | 7207 | 99.9% |
| G | A | 9246 | 7007 | 95.1% |
| T | C | 9305 | 9190 | 99.5% |
| A | C | 9554 | 10035 | 99.2% |
| A | G | 9599 | 11209 | 99.3% |
| C | A | 9654 | 11679 | 99.4% |
| T | A | 9822 | 11256 | 93.2% |
| A | G | 9878 | 11021 | 99.4% |
| C | T | 9966 | 11551 | 99.3% |
| T | C | 10049 | 11477 | 99.5% |
| T | C | 10264 | 8039 | 99.5% |
| C | T | 10346 | 5724 | 98.9% |
| A | G | 10562 | 7589 | 99.5% |
| G | A | 10843 | 6823 | 99.4% |
| T | C | 10887 | 5454 | 99.3% |
| A | G | 10892 | 5553 | 99.1% |
| G | A | 10898 | 5855 | 99.4% |
| T | C | 10940 | 7516 | 99.4% |
| G | A | 11060 | 9536 | 99.5% |
| G | A | 11145 | 10886 | 99.6% |
| A | G | 11223 | 10990 | 99.5% |
| C | T | 11270 | 10177 | 99.6% |
| A | G | 11502 | 8543 | 99.4% |
| A | T | 11546 | 8378 | 99.5% |
| T | C | 11706 | 7590 | 99.5% |
| T | C | 11738 | 7760 | 96.8% |
| C | T | 11996 | 9159 | 99.6% |
| A | G | 12128 | 8533 | 99.8% |
| **A** | **C** | **12193** | **8846** | **99.5%** |
| T | C | 12326 | 7485 | 99.5% |
| G | A | 12416 | 7142 | 99.1% |
| A | G | 12647 | 9262 | 97.6% |
| T | C | 12704 | 11501 | 97.4% |
| G | A | 12712 | 11574 | 97.4% |
| A | G | 12761 | 12093 | 97.1% |
| C | T | 12809 | 12804 | 96.8% |
| T | C | 12815 | 12965 | 96.9% |
| T | C | 12820 | 12983 | 96.8% |
| T | G | 12987 | 9372 | 97.3% |
| C | T | 13076 | 7396 | 99.9% |
| T | C | 13223 | 7634 | 99.9% |
| G | A | 13334 | 7972 | 99.6% |
| G | A | 13376 | 7814 | 99.9% |
| T | C | 13406 | 8382 | 99.9% |
| C | T | 13457 | 8219 | 100.0% |
| C | T | 13731 | 7586 | 98.4% |
| C | G | 13805 | 6915 | 99.8% |
| T | C | 13835 | 6890 | 99.9% |
| T | C | 13884 | 6928 | 99.8% |
| C | T | 13889 | 6806 | 99.8% |
| C | T | 13895 | 6922 | 99.9% |
| A | C | 14141 | 9558 | 99.9% |
| A | C | 14171 | 8947 | 99.9% |
| C | T | 14385 | 7418 | 99.9% |
| T | C | 14558 | 6178 | 99.9% |
| C | T | 14618 | 6618 | 99.2% |
| A | G | 14639 | 6804 | 99.8% |
| A | G | 14672 | 7001 | 99.8% |
| G | A | 14794 | 8375 | 99.8% |
| A | G | 14927 | 8242 | 99.9% |
| G | A | 15101 | 6576 | 99.8% |
| T | C | 15119 | 6438 | 99.9% |
| G | T | 15438 | 6826 | 99.9% |
| A | G | 15476 | 6671 | 99.9% |
| T | C | 15503 | 6268 | 99.9% |
| C | T | 15539 | 6634 | 99.9% |
| T | A | 15620 | 6099 | 99.9% |
| T | C | 15650 | 6022 | 99.9% |
| G | A | 15728 | 5405 | 99.9% |
| **G** | **A** | **15777** | **4856** | **99.9%** |
| C | G | 15946 | 21 | 71.4% |
