# Supplementary material for: Genomic characterization of peste des petits ruminants vaccine seed “45G37/35-k”, Russia
Source: Vet Res. 2022 Oct 8;53:79. doi: 10.1186/s13567-022-01099-w (PMC9548208; doi:10.1186/s13567-022-01099-w)
Supplement: Supplementary file 2 — Additional file 2. Distribution of nucleotide and amino acid differences separating the PPR vaccine strains Nigeria/75/1 (Nig75/1) and 45G37/35-k (FRCVM). [file 13567_2022_1099_MOESM2_ESM.docx]

**Additional file 2** **Distribution of nucleotide and amino acid differences separating the PPR vaccine strains Nigeria/75/1 (Nig75/1) and 45 G37/35-k (FRCVM).**

| Genome Region | Number of nucleotide differences | Amino acid differences with Nig75/1 |
| --- | --- | --- |
| N (CDS) | 25(8) | AA138 S >N  AA424 T > I  AA425 D >N  AA451 T >I  AA464 P >S  AA469 P >L  AA473 Q >P  AA477 L > V |
| N-P non-coding | 3 |  |
| P (CDS) | 14(6) | AA74 G>D  AA99 Q>L  AA138 A>T  AA139 N>D  AA305 Y>F  AA451 G>E |
| P-M non-coding | 2 |  |
| M (CDS) | 21(4) | AA5312 A>S  AA5315 K>R  AA5317 K>E  AA5330 L>V |
| M-F non-coding | 47 |  |
| F (CDS) | 21(6) | AA18 T>A  AA110 L>V  AA441 K>E  AA463 L>V  AA485 R>G  AA492 M>I |
| F-H non-coding | 1 |  |
| H (CDS) | 30(12) | AA20 N>T  AA27 K>R  AA192 A>T  AA212 M>I  AA239 N>T  AA311 R>S  AA316 I>R  AA342 S>T  AA476 V>A  AA502 R>S  AA534 R>S  AA535 K>E |
| H-L non-coding | 4 |  |
| L | 42(4) | AA1700 L>F  AA1836 S>N  AA2051 A>S  AA2164 D>N |
